# An individualized telephone-based care support program for rural family caregivers of people with dementia: study protocol for a cluster randomized controlled trial

**DOI:** 10.1186/s12877-021-02575-2

**Published:** 2021-11-04

**Authors:** Yao Wang, Lily Dongxia Xiao, Yu Yu, Rong Huang, Hui You, Minhui Liu

**Affiliations:** 1 Xiang Ya School of Nursing, Central South University, Changsha, Hunan Province China; 2 College of Nursing and Health Sciences, Flinders University, Adelaide, South Australia Australia; 3 Department of Psychiatry, Yale School of Medicine, New Haven, CT USA

**Keywords:** Family caregivers, People with dementia, Telephone-based, Care support, Rural area, Cluster RCT

## Abstract

**Background:**

There are about 9.5 million people with dementia in China. Up to 99% of them are cared for by their family caregivers. Family caregivers are confronted with considerable difficulties and challenges while providing care. They often experience high levels of emotional, physical, financial, and social burdens. Caregivers in rural areas experience an even higher level of burden compared to their counterparts in urban areas due to fewer health resources for dementia care. However, so far, no intervention study has been conducted to support family caregivers in rural areas of China. The aim of this proposed study is to adapt and evaluate an evidence-based and culturally-tailored individualized telephone-based care support (ITBCS) program for family caregivers of people with dementia in rural China.

**Methods:**

A cluster randomized controlled trial (RCT) will be conducted to evaluate the effectiveness of the ITBCS compared with usual care for Chinese rural family caregivers of people with dementia. A total sample of 168 rural family caregivers will be recruited. The intervention components consist of a 3-month intensive telephone-based care support intervention followed by telephone-based follow-up consultations for 6 months. The control group will receive usual care services available for them. Outcome measures include carers’ subjective burden, depressive symptoms, health-related quality of life, social support, caregiving self-efficacy, and care recipients’ difficult behaviours and competence in activities of daily living at 3, 9 and 15 months after baseline. The potential cost-effectiveness of the ITBCS compared with usual care will be assessed as well.

**Discussion:**

If effective, the ITBCS program can be adapted and used in rural areas of China as a blueprint to improve the quality of home-based care for people with dementia. Findings from the present study are significant for developing evidence-based dementia care policy in rural China.

**Trial registration:**

Chinese Clinical Trial Registry, ChiCTR2000038821, Registered 4 April 2020, http://www.chictr.org.cn/showprojen.aspx?proj=62268.

## Background

The number of people living with dementia in the world will rise to more than 131 million by 2050 [1]. It is estimated that the total global costs for dementia were US $957.56 billion in 2015. The costs will be US $2.54 trillion in 2030 [1, 2]. The average cost per people living with dementia per year (US $19,144.36) far exceeds China’s per capita disposable income [1, 2]. More than half (62%) of the total cost was attributable to the loss of formal labor from unpaid family caregivers [1–3].

Across the world, family members of people living with dementia constitute an invisible health system because they are the main source of care. This is especially true in China where dementia care services are underdeveloped, and the Chinese mainstream culture, traditional Confucianism, emphasizes that the care and support of the elderly is the responsibility of their family [4, 5]. In China, the overwhelming majority of people with dementia are cared for by family caregivers at home without external informal or formal supports [4, 5]. The availability of community and home-based support services is very limited. There are no community–based dementia care services in community health service centers. It is predicted that this situation is even worse in rural areas due to fewer health services and resources for dementia care, and more difficulties in accessing and utilizing those limited resources. Moreover, family caregivers in rural areas have poorer knowledge about dementia and feel more stigmatized than their urban counterparts [6, 7]. Close attention and concern should therefore be given to such a group in public health and health policy, and more adequate support and help need to be delivered to them [8, 9].

A body of evidence from developed countries has shown that individualized tailored support to caregivers can maintain caregivers’ mental and physical health, improve the quality of life for both care recipients and caregivers, and reduce institutionalization rates, which would have huge social and economic benefits [10, 11]. However, so far, no support intervention study has been designed or conducted for family caregivers of people with dementia in rural China. Moreover, implementing best practices in community health centers in rural areas is particularly challenging [12–14]. Firstly, there are many cases in which family caregivers have either no opportunity or no time to leave people with dementia alone at home and there are no care support groups nearby [15, 16]. Secondly, the ratio of health professionals to population is low, so delivering care support services in an individualized face-to-face manner is less possible [17, 18].

Rapid advancements in telephone technology have significantly revolutionized global connectivity. In most low- and middle- income countries, there is an increased usage of telephones with coverage of up to 90% of the population. Expansion of phone services into inaccessible and rural areas has made it a favorite means of communication [19]. In China, the latest data showed over 1.6 billion telephone lines with teledensity of 112 [20]. The widespread adoption of and people’s dependence on the telephone make it an attractive approach for delivering health-related interventions [21]. Compared to face-to-face meetings and other remote-intervention methods (e.g., internet), telephone-based interventions are advantageous because they can be easily accessible, made widely available without concern for technological barriers, are highly flexible in terms of intervention hours, and are low cost [22]. The explosive development and deep penetration of telephones make telephone-based interventions feasible in rural areas in China.

Based on a comprehensive literature review, we found that telephone-based multi-component caregiver support interventions have shown positive effects on care burden, social support, depressive symptoms, and caregiving self-efficacy of family caregivers of people with dementia [15, 23, 24]. However, evidence is limited to interventions conducted in large urban cities in developed countries. Little is known about the efficacy of telephone-based care support among families with dementia with low socioeconomic status or rural residency. Therefore, the primary purpose is to examine the efficacy of an individualized telephone-based care support (ITBCS) program on health outcomes of family caregivers and people with dementia compared to usual care in a real-world setting. This paper reports the development and study procedure of this culturally sensitive ITBCS program.

## Methods

### Study design

A cluster randomized controlled trial (RCT) design will be used to conduct the ITBCS program. The protocol was developed and guided by the SPIRIT checklist.

### Ethical considerations

The study was approved by the IRB of behavioral and nursing research in Xiang Ya School of Nursing of Central South University (Approval no. E2020141). All study-related data will be stored securely in Xiang Ya School of Nursing. All data collected during the present study will only be accessible to the researchers.

### Setting

The study will be conducted in Hanshou County of Hunan Province, Central South of China. It has a population density of 382.6 people per square kilometer. In 2018, the per capita disposable income of Hanshou County was 13,560 RMB, which was similar to the median per capita disposable income of all Chinese rural residents (13,066 RMB) [25, 26]. The ratio of health professionals in Hanshou County to population is about 1.42 per 1000, reflecting the average ratio of 1.40 per 1000 in rural China reported by WHO [27, 28]. The percentage of people aged over 60 in Hanshou County is 19.88%, which is slightly higher than the overall percentage of people aged over 60 in China (17.3%) [27, 28]. In rural areas, given the culture and traditions, older people are usually cared for by family caregivers.

### Participants

Eligible participants are defined as: 1) the primary family caregivers of a people diagnosed with dementia living at home; 2) aged 18 years or above; 3) having access to a reliable telephone; 4) having cared for people with dementia for no less than 6 months; 5) able to communicate in Mandarin, and 6) able to provide informed consent to participate in this study. The exclusion criteria for participants are: 1) hired caregivers by the family; and 2) with diseases that are unable to provide informed consent (e.g., severe mental health problems or cognitive impairment).

### Sample size

The sample size calculation for the study is based on the primary outcome, “caregiver burden score” measured by the 22-item Zarit Burden Interview (ZBI) scale, and is estimated on the basis of an earlier RCT with a telephone-based multicomponent intervention program for family caregivers of people with dementia [29]. A significant decline of 18.44 in the burden score in the intervention group at post intervention with polled standard deviation of 17.0 were reported. These values will be used to calculate the sample size in the present study. Since randomization will be conducted by community health service centers, the sample size is adjusted by the intra-class correlation coefficient (ICC) to take into account the design effect. The adjustment considered a higher ICC of 0.3 with a design effect of 3.7 for a cluster size of 10 participants in each center. To achieve at least 90% power at a 5% significance level, the required number of centers would be 7, with 70 family caregivers in each group. Assuming an attrition rate of 20%, we will need 84 participants per group or 168 in total.

### Recruitment

**Fig. 1:**
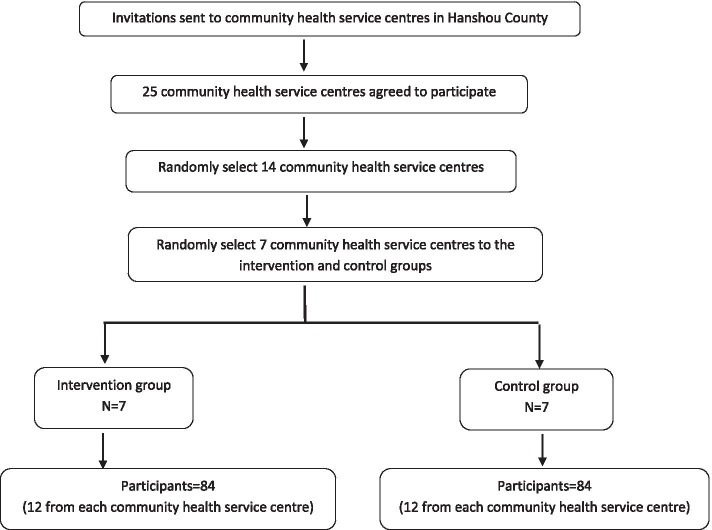
Flow-chart of sample framework and number of participants in the project

Potential participants will be recruited in two ways. Firstly, the health records of elderly people in each participating rural community health service center will be reviewed by a trained project field officer. Secondly, referrals from health professionals and family caregivers will be used as a strategy to recruit participants. Appropriate family caregivers will be notified of opportunities to join this program and a full explanation will be provided. Family caregivers who meet the inclusion criteria and would like to participate in the program will be contacted by a project field officer to set up an initial meeting. During the enrolment meeting, written materials about this program and their rights and risks as a participant will be provided and written informed consent will be obtained. All family caregivers will be informed that they can withdraw at any time. We will also document the reasons why caregivers refused to participate in the study. The research team will use a de-identifiable form to collect the information provided by participants. All collected information will be treated anonymously and confidentially. The selection process and the anticipated number of participants is shown in Fig. 1.

### Randomization and allocation concealment

A randomization protocol will be used to select fourteen community health service centers in Hanshou County randomly (http://stattrek.com/statistics/random-number-generator.aspx). There will be seven centers in each group (intervention group and control group). An external biostatistician who will not participate in the study intervention or data analysis will conduct the randomisation. All eligible family caregivers living in the intervention communities will be the intervention participants, while those living in the control communities will be control participants.

### Blinding

Because randomisation will occur at the level of individual centers, individual participants will not be blind to the condition they will receive. The researchers and statisticians responsible for data collection and analysis will be blinded regarding group assignments. Only the team member conducting the intervention will be informed of group assignments given the nature of the intervention.

### Intervention

Three theoretical approaches inform the ITBCS program. First, Ruth Cohn’s theme-centered interaction (TCI) model that emphasizes partnerships with family caregivers to improve their self-management of dementia at home and thereby reduce difficult behaviours of people with dementia [30]. Second, the perspective of systemic therapy that emphasizes psychological burdens should not just be considered at the individual level, but also at the system level where people with dementia are cared for [31]. Third, the principles of behavioral therapy point out that difficult behaviours of people with dementia could be triggered by the behaviour of their family caregivers [32]. Therefore, coaching caregivers to apply evidence-based care approaches can reduce difficult behaviours and caregiver burdens. The ITBCS program is designed to enhance family caregiver coping abilities through active problem-solving and facilitating positive changes within the family system via individual phone care support sessions.

Participants will be enrolled for 15 months. For intervention and control groups, there are four assessment time points: the beginning of the study (T-0) and at 3 months after baseline (T1), 9 months after baseline (T2), and 15 months after baseline (T-3). The assessment time points and intervention activities for both groups is shown in Table 1. The ITBCS program includes two major components: intensive ITBCS intervention and telephone consultations at the maintenance phase.

**Table 1:**
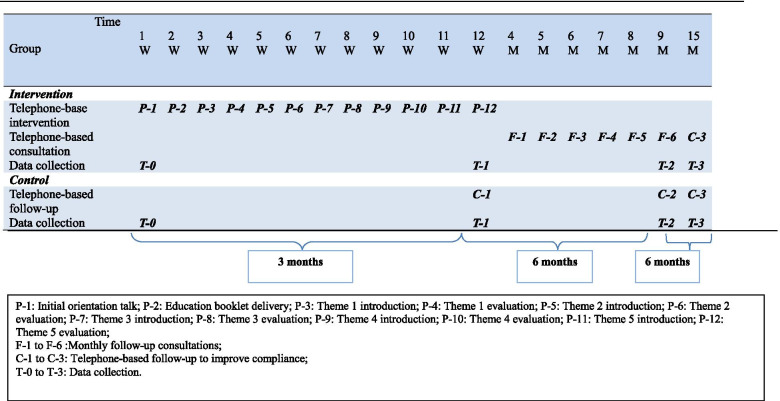
Intervention activities timeline

P-1: Initial orientation talk; P-2: Education booklet delivery; P-3: Theme 1 introduction; P-4: Theme 1 evaluation; P-5: Theme 2 introduction; P-6: Theme 2 evaluation; P-7: Theme 3 introduction; P-8: Theme 3 evaluation; P-9: Theme 4 introduction; P-10: Theme 4 evaluation; P-11: Theme 5 introduction; P-12: Theme 5 evaluation

F-1 to F-6: Monthly follow-up consultations

C-1 to C-3: Telephone-based follow-up to improve compliance

T-0 to T-3: Data collection

#### Intensive ITBCS intervention

Each family caregiver will receive 12 telephone contacts distributed over 3 months that focus on five care support themes. The objective and content of each theme is shown in Table 2.

**Table 2:** Care support theme and content

Theme	Objective	Content
1. Caregiver Burden	Provide knowledge about the negative influence of stress. Strengthen family caregivers’ skills in managing the care burden.	• Provide education on caregiving, stress, and safety. • Teach stress management strategies: for example, stretching exercises, breathing exercise.
2. Difficult behaviours	Enhance family caregivers’ ability to manage difficult behaviours.	• Provide knowledge about dementia symptoms. • Provide step-by-step strategies to manage specific difficult behaviours. • Conduct brainstorming of difficult behaviours management strategies.
3. Depression	Provide skills in mood management. Enhance family caregivers’ psychological well-being.	• Provide knowledge about the importance of psychological well-being. • Teach family caregiver how to engage in pleasant events. • Teach family caregiver how to manage mood. • Establish a schedule of pleasant events.
4. Self-care and healthy behaviours	Enhance family caregiver’s physical well-being and teach self-care behaviours	• Provide knowledge about preventive health practices and self-care. • Teach healthy behaviours (e.g., physical exercise, sleep, nutrition, adherence to prescribed medication)
5. Social support	Enhance family caregiver’s social support, and care support	• Provide knowledge about the importance of social support. • Teach family caregiver to access community resources and refer to resource guide. • Teach communication skills with health professionals and other family caregivers • Strengthen participation of telephone-based support sessions.

The intensive ITBCS intervention consists of four steps.

- *Step 1 Orientation talk:* In order to address the concerns of each family caregiver during the structured care support sessions (step 3), the project field officer should have an understanding of their particular care situation. Therefore, at the beginning of the intervention, the project field officer will conduct an orientation telephone conversation with each family caregiver (lasting around 30 min). In addition, the ITBCS program process and conversation rules will be discussed. There will be four project field officers in each center and each project field officer will be assigned three family caregivers.
- *Step 2 Education booklet delivery:* To support the thematic introduction of each care support session (step 3), the research team will develop an education booklet summarizing information on the five support themes and listing project field officer contact details for family caregivers. This booklet could also be used as a care diary during support sessions. Each participating caregiver will receive a copy.
- *Step 3 Structured care support sessions:* Each family caregiver will receive five telephone-based care support sessions delivered by a project field officer. Support sessions will be scheduled to occur every 2 weeks. The duration of a support session is about 60 min. At the beginning of a support session, one of five care support themes will be introduced and discussed for around 30 min. After the thematic discussion, the remaining 30 min will be available for a discussion between the project field officer and family caregiver in order to solve individual problems within this support theme and better utilise family resources. Table 3 outlines an exemplary structure of a care support session.
- *Step 4 Structured evaluation of each care support session:* In the week after each care support session, a project field officer will conduct a structured telephone-based interview with each family caregiver. The semi-structured questions include the following: What was good? What did you benefit from this session? What did you miss, and do any questions remain unanswered? Which issues do you want to add to the next session?

**Table 3:** An exemplary structure of a care support session

(1) Brief review of the previous session content and identification of any changes since then. Introduce the focus of this session and how it will be structured.	
(2) Detailed introduction of the care support theme.	
(3) Check and evaluate of usage of the information and strategies in the information booklet.	
(4) Provide training or education to help family caregivers solve problems and better use family resources.	
(5) Identify and initiate a problem-solving approach focusing on the caregiver’s individual problems and risk behaviours.	
(6) Closure: • briefly review problem areas. • briefly review strategies. • emphasise the importance of implementation strategies in daily care practice. • establish date and time of next session.	

#### Maintenance telephone consultation session

After the intensive ITBCS intervention, participants will receive monthly maintenance telephone consultation sessions for 6 months. A project field officer will conduct the maintenance telephone consultation. Each consultation lasts no more than 20 min.

#### Train the trainer

The current study uses a train the trainer model to increase intervention fidelity and build sustainability of the ITBCS program. Four project field officers in each center will be trained in the ITBCS intervention and carer support skills using a train the trainer model of carer support based on the facilitating guidebook developed by the research team. The responsibilities of project field officers will include: (1) participants recruitment; (2) providing ITBCS to participants; and (3) making reminder telephone calls for the four time-point assessments. The use of the facilitating guidebook will achieve standardization of the intervention delivery.

#### Treatment as usual for the control group

Participants in the control group will not receive the ITBCS program during the intervention phase. They will continue with usual services available for them during this period without any restriction (usual care). From our previous study in this population, very few family caregivers receive any support outside their family in rural China, so we do not expect this to influence the study outcome. Similar to the intervention group, One GPs and one RN from each community health service center in the control group will be trained as project field officers. Their responsibilities will include: (1) participants recruitment; (2) making telephone reminder calls for the four time-point measurements; and (3) delivering the ITBCS program to participants in the control group after completion of the present study. The participants will complete the same measurements at the same time points as the ITBCS group.

**Fig. 2:**
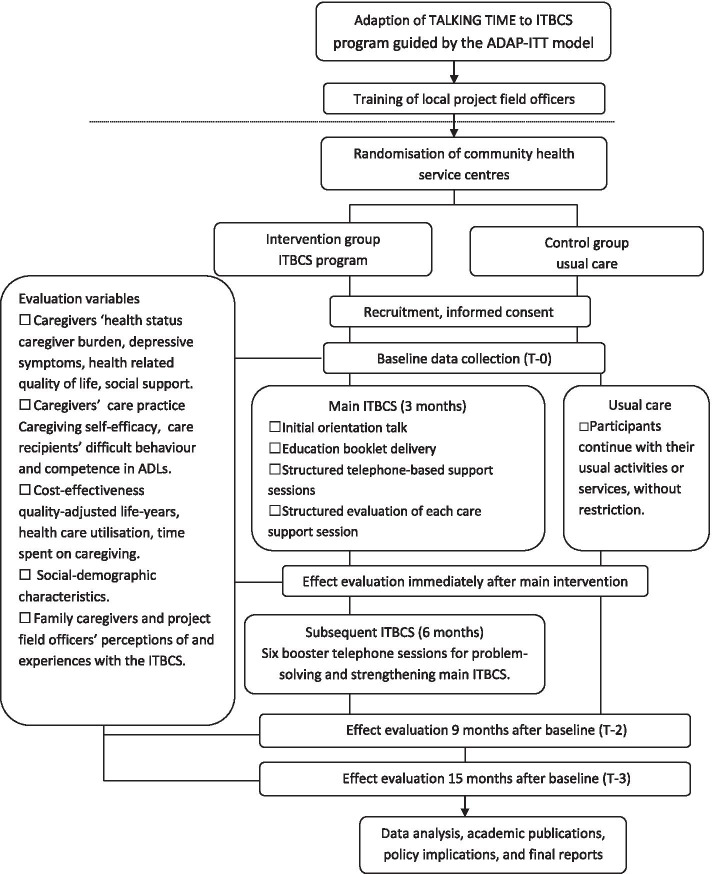
Flowchart of the study

Upon completing the 15-month study, all participants will receive a “thank you” letter for their participation in the study. The study flowchart is displayed in Fig. 2.

### Variables and measurements

Outcomes will be measured in both groups at baseline (T-0), immediately after main ITBCS (T-1), 9 months post-intervention (T-2), and 15 months post-intervention (T-3). Research assistants blinded to the group assignments will be responsible for collecting all the measures employed in the proposed study.

#### Primary outcomes

The primary outcome measure is family caregivers’ perceived burden at 15 months follow-up adjusted for baseline value.

#### Secondary outcomes

Secondary outcomes are the followings adjusted for baseline value: (i) family caregivers’ depressive symptoms; (ii) family caregivers’ social support; (iii) family caregivers’ caregiving self-efficacy; (iv) care recipients’ difficult behaviours; and (v) care recipients’ competence in activities of daily living.

#### Instruments

Table 4 shows the measurements and their relationship to outcomes and hypotheses of the proposed study.

**Table 4:** Instruments used for outcome assessment

Categories	Instruments description	Hypothesis & description
Primary outcome	Zarit Burden Interview (ZBI): This 22-item scale is used to assess the self-perceived burden by caregivers for providing care to impaired elderly. All items are related on a 5-point scale. The response range is 0 (never) to 4 always, with higher scores indicating higher perceived burden. Cronbach’s alpha of the Chinese version is 0.89 [33].	The ITBCS will reduce the subjective burden of family caregivers.
Secondary outcome	Centre for Epidemiologic Studies Depression Scale (CES-D): This 20-item scale measures the frequency of common depressive symptoms. The response range is 0 to 3. Higher scores indicate more severe depressive symptoms. Cronbach’s alpha of the Chinese version is 0.85 [34].	The ITBCS will reduce the depressive symptoms of family caregivers.
	12-item Short-Form Health Survey (SF-12): This 12 question instrument will be used to assess health-related quality of life by eight sub-scales. Cronbach’s alpha of the Chinese version is 0.910 [35].	The ITBCS will improve the health-related quality of life of family caregivers.
	Social Support Rating Scale (SSRS): This 10-item scale is used to assess objective support, subjective support, and utilization of support on a 7-point scale. A higher score indicates higher levels of social support. SSRS is in the Chinese version. Cronbach’s alpha is 0.94 [36].	The ITBCS will improve the social support of family caregivers.
	Revised Scale for Caregiving Self-Efficacy (RSCSE): This scale includes three subscales to assess caregivers’ beliefs about their ability to address caregiving challenges. The score ranges from 0 (cannot do at all) to 100 (certainly can do). Cronbach’s alpha of the Chinese version for the three subscales is 0.92,0.95 and 0.86 respectively [37].	The ITBCS will improve the self-efficacy of family caregivers in providing care.
	Neuropsychiatric Inventory-Q (NPI-Q): This scale is used to measure the severity of behavioural and psychological symptoms of dementia (BPSD). A higher score indicates the more severe the BPSD and the higher caregiver distress level. Cronbach’s alpha of the Chinese version is 0.851 [38].	The ITBCS will reduce the difficult behaviours of people with dementia.
	Disability Assessment for Dementia (DAD): This scale is used to assess functional disability in people with dementia. Through an interview, caregivers are required to indicate the actual performance of the people with dementia in functional tasks over the previous 2 weeks with responses of “no”, “yes”, or “not applicable”. A higher score indicates less functional disability while a lower score indicates more dysfunction. Cronbach’s alpha of the Chinese version is 0.91 [39].	The ITBCS will improve competence in ADLs of people with dementia.

#### Covariates

Socio-demographic information about family caregivers and people with dementia will be collected at baseline through a self-designed demographic information sheet which includes the caregiver’s gender, age, employment status, relationship to the care recipient, co-residence with care recipient or not, education background, caring duration and hours spent on care activities. Demographic questions will also ask about the care recipient’s gender, age, duration of dementia, health insurance and living assistance.

#### Economic evaluation

- ***Quality-adjusted life-years (QALYs):*** The SF-12 will be used to collect data for the QALYs calculation. QALYs will be calculated using the SF-6D preference to estimate the utilities based scoring algorithm developed by Brazier and colleagues [40].
- ***Health care utilization:*** This proposed study will measure the direct costs related to the family caregiver’s situation and people with dementia. Direct costs will include medications, admissions to hospitals, admission to other institutions, and the number of consultations with health professionals (GP, RN, medical specialist, physiotherapist, etc.). Indirect costs are reduced efficiency at work (presenteeism) and the productivity loss caused by work absenteeism.
- ***Time spent on caregiving:*** Caregivers will be asked to estimate the total hours spent on providing care for people with dementia in the past week. In addition to this, information of the availability of other informal caregivers will be collected by the research team. The total time the primary family caregiver and other informal caregivers spent on providing care will be recorded and calculated.

### Intervention fidelity

The ITBCS project field officers will receive 12 hours of training to standardize delivery of the ITBCS program prior to participant recruitment. The research team will support and supervise the project field officers while implementing the ITBCS program. To maintain the intervention fidelity, records will be kept by both project field officers and family caregivers of people with dementia including: (1) intervention record-project field officer version; and (2) intervention record-family caregiver version. Compliance with the intervention protocol will be checked by using these two tools. In addition, the study will have a safety officer (SO). The responsibilities of the SO include, but are not limited to, auditing study processes, maintaining data confidentiality and safety, and contingency planning.

### Statistical methods

All data will be double entered into an Excel database on two separate occasions and exported to SPSS software 25.0 for analysis. Internal consistency of the seven instruments will be tested using Cronbach’s alpha coefficient. For the pilot study, paired *t* tests will be conducted to evaluate whether any differences occurred in participants on the seven outcome variables pre- and post-intervention. The research team will perform all analyses of the cluster RCT study based on the intention to treat principle. Baseline data between the experimental and control group will be compared. Missing data will be imputed if deemed necessary. The most appropriate imputation methods will be decided based on the amount and pattern of missing data. A mixed effect model, which takes into account the repeated measurement of outcomes, will be conducted to analyse the primary outcome. The mixed effect model will include treatment, time, and interaction between time and treatment as the fixed effect, baseline measurement of outcome as the covariate, and subject as a random effect. The treatment difference and its 95% confidence interval (CI) will be derived from the mixed effect model. Moreover, adjusted treatment difference will be derived by adding the pre-specified covariates into the mixed effect model. All other outcomes (CES-D, SF-12, SSRS, RSCSE, NPI-Q and DAD) will be analyzed separately using this general framework.

Cost-effectiveness analyses (CEA) and cost-utility analyses (CUA) will be conducted. The difference of total costs among scenarios will first be calculated, which will be further divided by the difference in average effect sizes to estimate the incremental cost-effectiveness ratio. Inflation correction is not necessary as we will evaluate the costs covering 9 months. Multiple imputation methods will be applied to deal with the missing data in the follow-up measurements. A 95% confidence interval of the mean difference in the treatment groups’ total costs will be calculated. Cost-effectiveness acceptability curves will indicate the cost-effectiveness comparison between the ITBCS program and the usual care, with a range of different ceiling ratios presenting decision uncertainties. Furthermore, sensitivity analyses to test the robustness, such as scenarios that vary key variables will be conducted.

### Trial status

Most of the field research activities of our team have been restricted because of the COVID-19, which has delayed the project implementation. The paricipants recruitment will start by the end of December 2021.

## Discussion

It is urgent to develop feasible and cost-effective programs that satisfy the needs of family caregivers of people with dementia across the course of dementia in rural areas in China, improving quality of life and health-related outcomes for family caregivers and care recipients [3, 6, 7]. Effective interventions tailored to rural health resources and rural family caregivers’ knowledge and skills, education level, and cultural values are much needed in dementia care to generate research evidence to inform policy, resource, and practice development in dementia care in rural China. To our knowledge, this study is the first to examine the efficacy and cost-effectiveness of an evidence-based ITBCS through a cluster RCT in China.

The development of the ITBCS program is based on the successful telephone-based multi-component intervention (TALKING TIME) which is designed to enhance family caregiver coping abilities through facilitating positive changes and active problem-solving within the family system via individual phone care support sessions [15, 32]. This proposed study will be the first to adapt TALKING TIME into the Chinese context and to examine the feasibility, efficacy, and cost effectiveness of this program through a cluster RCT in Central South China. The findings are expected to contribute to the development of dementia health care services in China.

There will be a number of challenges when implementing the trial. Community health professionals in rural China are less educated and skilled than their counterparts in urban cities. In such a situation, the project field officers will be supported in the project by skilled professionals to ensure scaling-up of the present study and future sustainability. The training materials and information booklet will be developed to ensure easy understanding. The project team will train a replacement project field officer if one is unable to continue their role due to unforeseen reasons. A WeChat group will be set up for each group of project field officers to raise concerns and ask questions. The project team will be available to answer questions and provide advice regarding ITBCS delivery. Another challenge is how to ensure high compliance with the intervention and retain participants. Multiple strategies will be applied to maximize compliance in this population, for example, the information booklet, reminder calls, telephone consultations, and cash-based gifts as incentives. Despite many challenges, if proved effective, this study would provide the results of the efficacy of the ITBCS program and be a basis for the ITBCS program dissemination to other rural communities and thereby reducing the disease burden of dementia in China.

### Limitations

This proposed study has some limitations. Firstly, the reliance on self-report data and.

the limited geographical reach of the present proposed study limits its generalizability.

Any long-term effects of the ITBCS program on the outcomes will be difficult to show because of the limited timeframe of this proposed project. However, the sample size calculated is theoretically sufficient to detect a meaningful statistical difference. We also use the cluster randomization technique to maximize population representation and increase follow-up to 12 months after intervention completion. We have also included health outcomes of people with dementia to minimize the self-report bias of family caregivers.

### Significance

The WHO urges all countries to make dementia a public health priority, develop programs to improve family caregivers’ and care recipients’ quality of life, and provide equitable support for family caregivers across rural and urban areas [8, 9]. The proposed study aims to develop and provide appropriate and meaningful individualized care support for family caregivers of people with dementia in rural China in a cost-effective way. If proved effective, the present proposed interdisciplinary study will potentially improve the quality of life and health outcomes for millions of people with dementia and their family caregivers. Moreover, in addition to the benefits from the findings, our study provides feasible solutions for intervention program maintenance by building local capacity. The rural community health professionals selected as local project field officers will be trained in dementia care intervention skills, which will facilitate the dissemination of the dementia care intervention program to other communities in the future using a train-the-trainer model.

## Data Availability

All data collected during the present study will only be accessible to the researchers. Public access to the study-related data will be available upon approval via the Chinese clinical trial registry.

## Abbreviations

BPSD: Behavioral and psychological symptoms of dementia
CEA: Cost-effectiveness analysis
CES-D: Centre for Epidemiologic Studies depression scale
CI: Confidence interval
CUA: Cost-utility analysis
DAD: Disability assessment for dementia
ICC: Intra-class correlation coefficient
ICERs: Incremental cost-effectives ratios
ITBCS: Individualized telephone-based care support
SSRS: Social support rating scale
NPI-Q: Neuropsychiatric inventory-Q
QALYs: Quality-adjusted life-years
RCT: Randomized controlled trial
RSCSE: Revised scale for caregiving self-efficacy
SF-12: 12-Item short-form health survey
TCI: Theme-centered interaction
ZBI: Zarit burden interview
